# Host-derived extracellular RNA promotes adhesion of *Streptococcus pneumoniae* to endothelial and epithelial cells

**DOI:** 10.1038/srep37758

**Published:** 2016-11-28

**Authors:** Dariusz Zakrzewicz, Simone Bergmann, Miroslava Didiasova, Benedetto Daniele Giaimo, Tilman Borggrefe, Maren Mieth, Andreas C. Hocke, Guenter Lochnit, Liliana Schaefer, Sven Hammerschmidt, Klaus T. Preissner, Malgorzata Wygrecka

**Affiliations:** 1 Department of Biochemistry, Faculty of Medicine, Universities of Giessen and Marburg Lung Center, Friedrichstrasse 24, 35392 Giessen, Germany; 2 Department of Medical Microbiology, Helmholtz Centre for Infection Research, Inhoffenstrasse 7, 38124 Braunschweig, Germany; 3 Department of Internal Medicine/Infectious Diseases and Pulmonary Medicine, Charité-University Medicine Berlin, Chariteplatz 1, 10117 Berlin, Germany; 4 Institute of Pharmacology and Toxicology, Goethe University School of Medicine, University Hospital, Theodor Stern Kai 7, 60590 Frankfurt am Main, Germany; 5 Department Genetics of Microorganisms, Interfaculty Institute for Genetics and Functional Genomics, Ernst-Moritz-Arndt Universität Greifswald, Friedrich-Ludwig-Jahn-Strasse 15a, 17489 Greifswald, Germany; 6 German Centre for Lung Research, Giessen, Germany.

## Abstract

*Streptococcus pneumoniae* is the most frequent cause of community-acquired pneumonia. The infection process involves bacterial cell surface receptors, which interact with host extracellular matrix components to facilitate colonization and dissemination of bacteria. Here, we investigated the role of host-derived extracellular RNA (eRNA) in the process of pneumococcal alveolar epithelial cell infection. Our study demonstrates that eRNA dose-dependently increased *S. pneumoniae* invasion of alveolar epithelial cells. Extracellular enolase (Eno), a plasminogen (Plg) receptor, was identified as a novel eRNA-binding protein on *S. pneumoniae* surface, and six Eno eRNA-binding sites including a C-terminal 15 amino acid motif containing lysine residue 434 were characterized. Although the substitution of lysine 434 for glycine (K434G) markedly diminished the binding of eRNA to Eno, the adherence to and internalization into alveolar epithelial cells of *S. pneumoniae* strain carrying the C-terminal lysine deletion and the mutation of internal Plg-binding motif were only marginally impaired. Accordingly, using a mass spectrometric approach, we identified seven novel eRNA-binding proteins in pneumococcal cell wall. Given the high number of eRNA-interacting proteins on pneumococci, treatment with RNase1 completely inhibited eRNA-mediated pneumococcal alveolar epithelial cell infection. Our data support further efforts to employ RNAse1 as an antimicrobial agent to combat pneumococcal infectious diseases.

*Streptococcus pneumoniae* is a Gram-positive bacterium, which is a main cause of community-acquired pneumonia (CAP). Initial treatment of CAP mostly includes antibiotic therapies^1^. However, pneumococcal antibiotic resistance has escalated dramatically over the last three decades making pneumonia a leading cause of death, especially among high-risk groups such as children under the age of five, elderly people, and immunocompromised individuals^2^,^3^. An increasing number of penicillin and macrolide resistant isolates and also a continuous increase in multidrug resistance (MDR, resistant to ≥ 3 classes of antimicrobials) have been reported^4^. The invention of a 13^th^ valent conjugate vaccine (Prevnar13^®^) has generated limited protection in children^5^. Due to the restricted serotype coverage combined with the low vaccination status of the elderly and immunocompromised patients^5^, novel strategies against this pathogen are sorely needed.

In addition to the pore-forming cytotoxin pneumolysin and the phagocytosis-inhibiting polysaccharide capsule, the virulence of *S. pneumoniae* is promoted by the capacity of bacteria to bind to and internalise into host cells and to spread into host tissue. All these processes require the involvement of bacterial cell wall-associated components, adhesins^6^. Adhesins bind to eukaryotic cell surface receptors^7^,^8^ or extracellular matrix (ECM) components^9^,^10^. They can be divided into two groups: cell-wall-anchored polypeptides^8^,^11^,^12^ and anchorless proteins^13^,^14^,^15^,^16^,^17^. The last group is represented, among others, by the glycolytic enzyme enolase (Eno). Extracellular Eno is a surface-located plasminogen (Plg)-binding protein of *S. pneumoniae*^17^,^18^. It possesses two Plg-binding sites, one comprising C-terminal lysine residues (^433^KK^434^) and the second involving the amino acid sequence ^248^FYDKERKVY^256^ 17,18,19. The importance of Eno in the binding of Plg to bacterial surface was underscored by the reduced invasive potential of pneumococcal mutants expressing Eno with amino acid substitution in both Plg-binding motifs^19^.

Accumulating evidence suggests that intracellular macromolecules such as proteins and nucleic acids are released into the extracellular milieu where they may serve as alarmins and thus affect various host defence processes^6^,^20^. Extracellular nucleic acids were found to induce humoral and cellular immune responses during infection and to promote the formation of fibrin that entraps invading microbes^20^,^21^,^22^. In particular, host-derived eRNA was reported to: i) enhance fibrin deposition by the activation of the contact phase pathway^22^,^23^, ii) increase the adhesion and transmigration of leukocytes to and across the endothelium^24^, iii) and, augment the release of proinflammatory cytokines such as macrophage inflammatory protein-2, interleukin-1β, −6, −10, and tumor necrosis factor α from neutrophils, bone marrow-derived macrophages and endothelial cells^24^,^25^,^26^,^27^. However, the direct interaction of host-derived eRNA with pneumococcal surface, and the potential consequences of this process on bacterial tropism have not been addressed thus far. Here, we propose that the eRNA facilitates bacterial adherence and invasion by serving as a “bridging molecule”, which connects bacterial cell surface-associated proteins with components of the host cell membrane. Consequently, administration of RNase1 can disrupt the invasion process and serves as a new anti-infectious agent.

## Results

### Extracellular RNA promotes pneumococcal infection of lung epithelial cells

**Figure 1:**
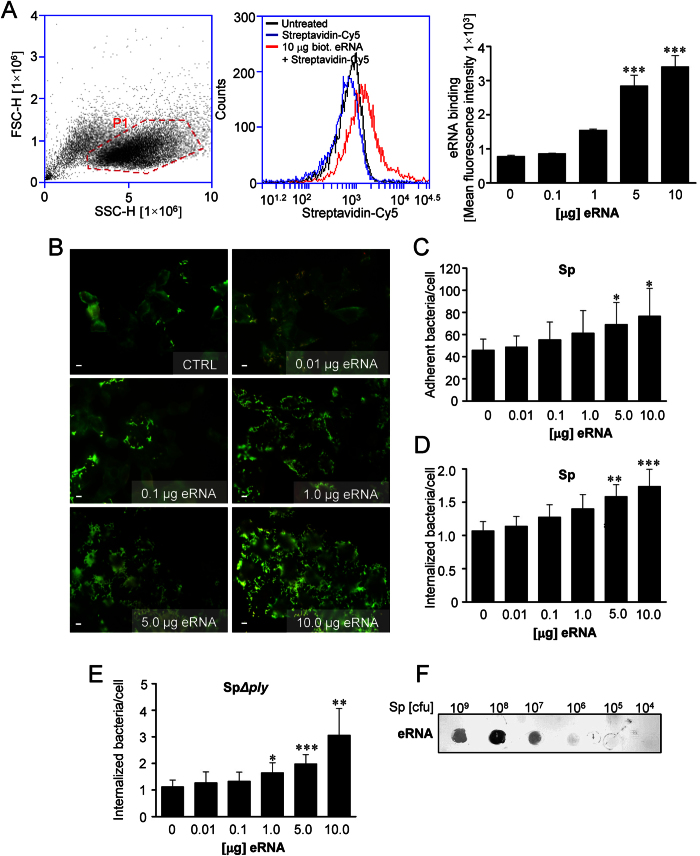
Pneumococcal infection is attenuated in the presence of eRNA. (**A**) A549 cells were incubated with different concentrations of biotinylated eRNA. After eRNA treatment cells were analysed by flow cytometry. Gate based on forward and side scatter was set to eliminate cellular debris and cell clusters (left panel). The binding of eRNA to cell membrane was analyzed in the Cy5 channel of the flow cytometer. Histogram overlay illustrates control cells (black), and cells incubated with 10 μg biotinylated eRNA (red) or Cy5-conjugated Streptavidin (blue; middle panel). Dose-dependent binding of eRNA to A549 cells analyzed by flow cytometry (right panel). Data represent mean values (±SEM). n = 4, ***p ≤ 0.001 *vs* control (Ctrl). (**B**) Human lung pneumocytes A549 were preincubated with different doses of eRNA (0.01–10 μg) and pneumococcal host-cell adherence and internalization of serotype 2 *S. pneumoniae* (Sp)-strain (ATCC11733) were measured by immunofluorescence staining and microscopy. The staining procedure resulted in Alexa568-labeled intracellular bacteria (red fluorescence) and Alexa488/568-labeled extracellular pneumococci (green/yellow). Scale bars in the images represent 10 μm. (**C**) Quantification of pneumococcal adherence to A549 cells after treatment with different doses of eRNA. Data represent mean values ± SEM; n = 3; *p ≤ 0.05. (**D**) Quantification of pneumococcal internalization into A549 cells after eRNA treatment. Data represent mean values ± SEM; n = 3; **p ≤ 0.01; ***p ≤ 0.001. (**E**) The internalization of *S. pneumoniae* pneumolysin-deficient strain (SpΔ*ply*) into A549 cells measured by antibiotic protection assay. Data represent mean values ± SD; n = 3; *p ≤ 0.05, **p ≤ 0.01; ***p ≤ 0.001. (**F**) Different amounts of *S. pneumoniae* (10^4^, 10^5^, 10^6^, 10^7^, 10^8^ and 10^9^ cfu) were immobilized on the nitrocellulose membrane. The binding of biotinylated eRNA to bacteria was detected using peroxidase-coupled streptavidin.

In order to investigate the role of eRNA in *S. pneumoniae* infection, we first tested whether eRNA associates with lung epithelial cell membrane. To this end, A549 cells were incubated with the different concentrations of biotinylated eRNA and its interaction with the cell membrane was examined by FACS. Flow cytometry analysis revealed a dose-dependent binding of eRNA to epithelial cells (Fig. 1A). Next, to study the impact of eRNA on *S. pneumoniae* invasion of lung epithelial cells, adherence of bacteria to the cells in the absence or presence of eRNA was monitored. Preincubation of A549 cells with eRNA nearly doubled bacterial adhesion to A549 cells in a dose-dependent manner from 46.03 ± 9.9 adherent bacteria per cell without eRNA up to 76.64 ± 25 bacteria per cell with 10 μg eRNA (Fig. 1B,C). In general, the internalization rate of pneumococci into A549 cells was low, however in the presence of eRNA this process was enhanced too (Fig. 1D). Similar results were obtained when human umbilical vein endothelial cells (HUVEC) and human pulmonary microvascular endothelial cells (HPMEC) were employed (Supplementary Fig. S1). In order to clarify the effect of eRNA-supplementation on bacterial internalization, antibiotic protection assays with a pneumolysin-deficient strain (Sp*Δply*) were performed (Fig. 1E). Quantification of internalized bacteria by determination of cfu after plating revealed a three-fold increase in pneumococcal internalization from 1.1 ± 0.3 bacteria per cell up to 3.1 ± 1.0 bacteria per cell (Fig. 1E). Of note, no replication of bacteria during infection of A549 cells was observed (Supplementary Fig. S2). To test specificity of eRNA impact, human extracellular DNA (eDNA) was utilized, however, no effect of eDNA on bacteria invasion was observed (data not shown). To determine whether eRNA may directly bind to *S. pneumoniae*, bacteria (10^4^–10^9^ cfu) were immobilized on a membrane and incubated with biotinylated eRNA. As depicted in Fig. 1F, eRNA interacted with pneumococci in a dose-dependent manner. Interestingly, eRNA interacted not only with Gram-positive (*S. pneumoniae 35 A, S. pneumoniae TIGR4, S. pyogenes, Staphylococcus aureus* and *Listeria monocytogenes)*, but also with Gram-negative (*Neisseria meningitides* and *Escherichia coli)* bacteria and the binding of eRNA to bacteria was independent on the expression of pneumolysin (Sp35A*Δply*) (Supplementary Fig. S3). Altogether, these results indicate an important role of eRNA as a mediator of pneumococcal adhesion.

### Pneumococcal Eno interacts with extracellular nucleic acids

**Figure 2:**
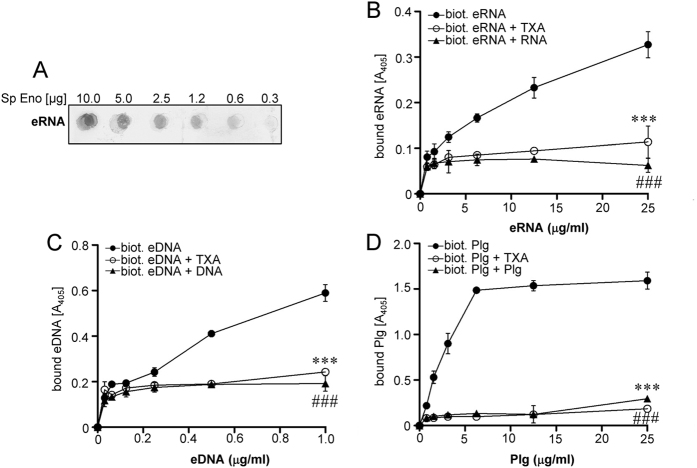
Extracellular RNA interacts with pneumococcal Eno. (**A**) Different amounts of recombinant *S. pneumoniae* Eno (0.32, 0.65, 1.25, 2.5, 5 and 10 μg) were spotted on a nitrocellulose membrane. The Eno/eRNA interaction was tested using biotinylated eRNA and peroxidase-coupled streptavidin. (**B**) Five μg of *S. pneumoniae* Eno were immobilized onto a microtiter plate and incubated with different concentration of biotinylated eRNA (●) in the presence or absence of unlabeled eRNA (▲) and tranexamic acid (TXA) (○). (**C**) Eno was incubated with different concentration of biotinylated eDNA (●) in the presence or absence of unlabeled eDNA (▲) and tranexamic acid (TXA) (○). (**D**) Eno was incubated with different concentrations of biotinylated plasminogen (Plg) (●) in the presence or absence of unlabeled Plg (▲) and TXA (○). Data represent mean values ± SEM; n = 3; ^###^p ≤ 0.001; ***p ≤ 0.001 *vs* biotinylated eRNA/eDNA/Plg (●).

A strong negative charge density associated with the RNA phosphate backbone favours interaction with positively charged protein domains^28^. Considering that bacterial cell wall-associated Eno possesses a highly positively charged (5.9 at pH 7.0) C-terminal region (K^405^-K^434^)^17^,^29^, we hypothesized that extracellular Eno becomes directly involved in the binding of eRNA to pneumococci. Indeed, the dot blot analysis revealed dose-dependent binding of biotinylated eRNA to bacterial Eno (Fig. 2A). The solid-phase binding assay employing immobilized Eno confirmed a dose-dependent interaction between Eno and eRNA (Fig. 2B). Furthermore, the binding of biotinylated eRNA to Eno was completely abrogated when competing unlabeled eRNA was added in a 100-fold molar excess or when the lysine analog tranexamic acid (TXA) was applied (Fig. 2B). Interestingly, an association of Eno with eDNA was observed as well (Fig. 2C). Similar to eRNA, the binding of biotinylated eDNA to Eno was abolished when competing unlabeled eDNA was added in a 100-fold molar excess or when the TXA was utilized (Fig. 2C). Plg was used as a positive control for binding to Eno (Fig. 2D).

### The Eno/nucleic acid interaction depends on the size of the nucleic acid fragments and on their nucleotide composition

**Figure 3:**
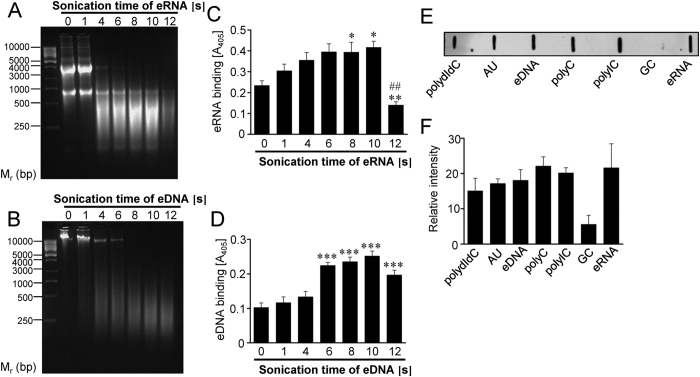
Eno/eRNA interaction depends on the size of the nucleic acids and on their nucleotide composition. (**A,B**) Total RNA and DNA were isolated from human A549 cells and subjected to different sonication times (1–12 s). Resulted eRNA (**A**) and DNA (**B**) was resolved on the agarose gel. (**C**) The binding of eRNA to pneumococcal Eno was determined using the solid-phase binding assay. Five μg of immobilized Eno were incubated with 10 μg sonicated eRNA. Eno/eRNA complexes were detected by chemiluminescence. Data represent mean values ± SEM; n = 3; **p ≤ 0.01; *p ≤ 0.05 *vs* control; ^##^p ≤ 0.001 *vs* 10 s of sonication. (**D**) The binding of eDNA to pneumococcal Eno was determined using the solid-phase binding assay. Five μg of immobilized Eno were incubated with 10 μg sonicated and biotinylated eDNA. Eno/eDNA complexes were detected by chemiluminescence. Data represent mean values ± SEM; n = 3; ***p ≤ 0.001 *vs* control. (**E**) Five  μg of synthetic oligonucleotides (polydIdC, AU, polyC, polyIC, GC), eRNA and eDNA were immobilized on the nitrocellulose membrane and their binding ability to biotinylated pneumococcal Eno was tested by dot blot. (**F**) Densitometry of (**E**). Data represent mean values ± SEM; n = 3.

In order to investigate whether the size of nucleic acid fragments may determine their binding ability to pneumococcal Eno, the nucleic acids were subjected to sonication (Fig. 3A,B). The maximal binding of eRNA to Eno was observed when eRNA was sheared to less than 500 ribonucleotide bases in length. Strikingly, longer sonication procedure generated smaller eRNA fragments that did not bind Eno effectively (Fig. 3C). In regard to eDNA, the strongest interaction of eDNA with Eno was observed, when eDNA fragments were no longer than 500 deoxyribonucleotide bases (Fig. 3D). Next, to test the impact of nucleotide composition on the binding of nucleic acids to Eno, synthetic oligonucleotides (polydIdC, AU, polyC, polyIC, GC) were applied. A filter-binding assay revealed association of Eno with polydIdC, AU, polyC, polyIC, eRNA and eDNA (Fig. 3E,F). The binding of Eno to GC was also observed, however, it was less pronounced (Fig. 3E,F). Ethidium bromide staining demonstrated equal immobilisation of oligonucleotides on a nylon membrane (data not shown).

Altogether, our data indicate that the binding of Eno to extracellular nucleic acids depends on their size and nucleotide composition.

### *S. pneumoniae* Eno possess multiple eRNA-binding sites

**Figure 4:**
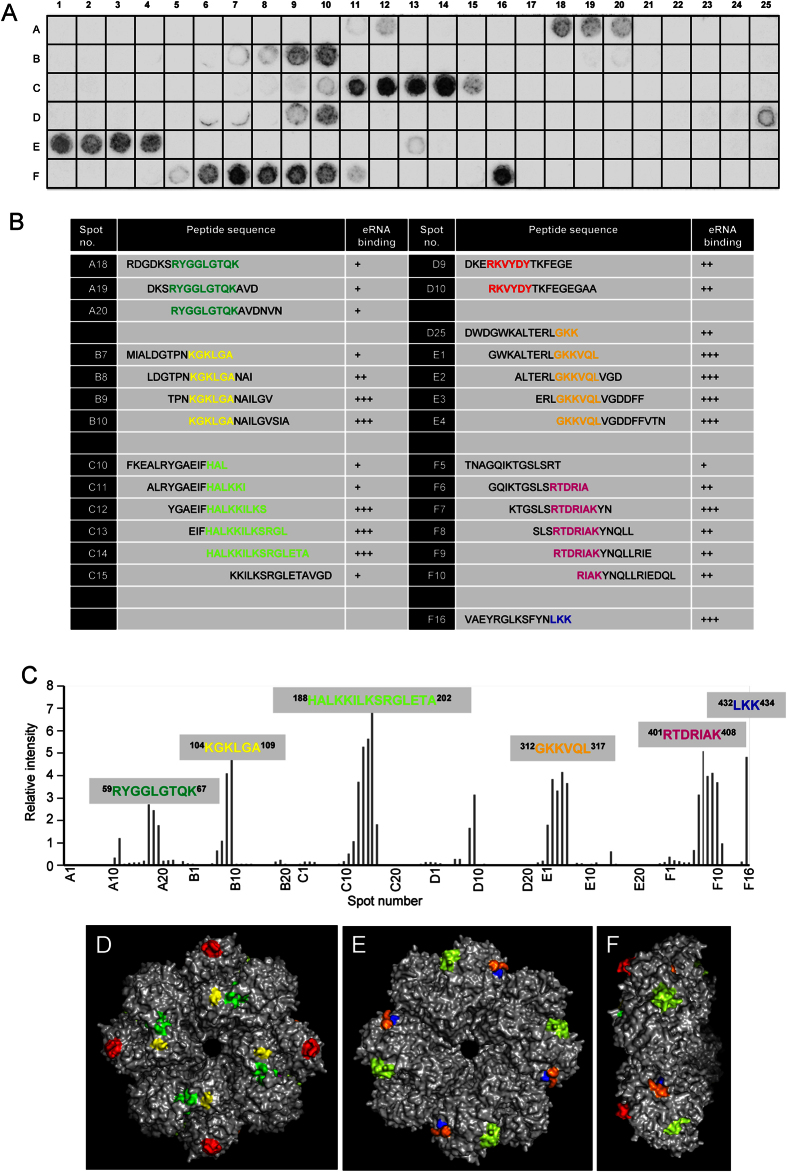
Identification and localization of eRNA-binding regions of Eno protein. (**A**) The entire pneumococcal Eno protein was divided into 141 overlapping peptides, each consisting of 15 amino acids, with an offset of three amino acids. The synthetic peptides were assayed for their ability to bind eRNA using dot blot analysis. (**B**) Sequences of spots (A18-A20, B7-B10, C10-C15, D9-D10, D25-E4, F5-F10 and F16) and the binding reactivity to biotinylated RNA. +, weak binding; ++, moderate binding; +++, strong binding. (**C**) Densitometry of (**A**). (**D–F**) Extracellular RNA-binding motifs on the surface of the pneumococcal Eno octamer. Six eRNA-binding regions in the Eno octamer are depicted in green (^59^RYGGLGTQK^67^), yellow (^104^KGKLGA^109^), light green (^188^HALKKILKSRGLETA^202^), orange (^312^GKKVQL^31^), magneta (^401^RTDRIAK^408^) and blue (^432^LKK^434^). Front view (**D**), backside view (**E**) and side view (**F**). Internal Plg-binding motif of Eno (^248^FYKERKVY^256^) is depicted in red. The figure was generated using PyMol (DeLano Scientific LLC).

In order to map the eRNA-binding sites, the entire pneumococcal Eno sequence was divided into 141 overlapping synthetic peptides, each consisting of 15 amino acids, with an offset of three amino acids^19^. The synthetic peptides were assayed for their capability to bind eRNA (Fig. 4A). The dot blot analysis using biotinylated eRNA revealed six potent eRNA-binding motifs: ^59^RYGGLGTQK^67^, ^104^KGKLGA^109^, ^188^HALKKILKSRGLETA^202^, ^312^GKKVQL^317^, ^401^RTDRIAK^408^ and ^432^LKK^434^ within Eno sequence (Fig. 4A–C). Interestingly, ^401^RTDRIAK^408^ and ^432^LKK^434^ motifs overlap with the C-terminal Plg-binding region^17^. Aforementioned peptides contain positively charged lysine and arginine residues contributing to their net positive charge ranging from 1.9 to 2.9. Next, the Protein Data Bank entry 1W6T was used to localize the identified eRNA-binding motifs in each monomer of the octamer structure of the pneumococcal Eno^30^. The putative eRNA-binding sequences were found to be distributed on each of the eight monomers of the Eno octamer on both, the front site and the backside (Fig. 4D–F). In line with previously published observations^30^, ^401^RTDRIAK^408^ and ^432^LKK^434^ motifs were found to be hidden in a structure of the protein and thus poorly accessible (Fig. 4D–F). Nevertheless, the eRNA-binding motif ^312^GKKVQL^317^ was found in close proximity to the C-terminal located lysines and thus may expand the eRNA-interacting surface (Fig. 4D,E).

### Mutation of the Eno C-terminal lysine 434 to glycine reduces binding of Eno to eRNA

**Figure 5:**
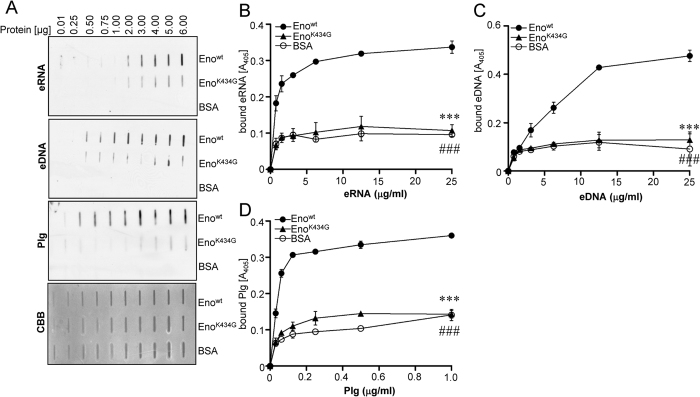
Mutation of Eno C-terminal lysine 434 reduces Eno-eRNA/eDNA interaction. (**A**) Bovine serum albumin (BSA), wt pneumococcal Eno (Eno) and Eno mutant (Eno^K434G^) were spotted on the nitrocellulose membrane. The binding of proteins to eRNA, eDNA and Plg was tested by dot blot. Commassie brilliant blue (CBB) staining was used as a loading control. (**B**) The binding of pneumococcal Eno^wt^ (●), Eno^K434G^ (▲) or BSA (○) to eRNA was determined using the solid-phase binding assay. Immobilized proteins were incubated with different concentration of biotinylated RNA. Eno/eRNA complexes were detected by chemiluminescence. (**C**) The binding of pneumococcal Eno^wt^ (●), Eno^K434G^ (▲) and BSA (○) to eDNA was determined using the solid-phase binding assay. The immobilized proteins were incubated with different concentration of biotinylated eDNA. Eno/eDNA complexes were detected by chemiluminescence. Data represent mean values ± SEM; n = 3; ^###^p ≤ 0.001; ***p ≤ 0.001 *vs* wt Eno (●). (**D**) The binding of Eno^wt^ (●), Eno^K434G^ (▲) or BSA (○) to Plg was determined using the solid-phase binding assay. Immobilized proteins were incubated with different concentration of biotinylated Plg. Eno/Plg complexes were detected by chemiluminescence. Data represent mean values ± SEM; n = 3; ^###^p ≤ 0.001; ***p ≤ 0.001 *vs* wt Eno (●).

Regardless of their accessibility, C-terminal lysine residues of pneumococcal Eno seem to play a significant role in the recruitment of bacterial surface proteolytic activity and thus in host tissue invasion *in vivo*^17^,^31^,^32^. Hence, we investigated whether a mutation of lysine residue 434 within Plg-binding site of Eno may impair also its association with nucleic acids. A slot-blot filter binding assay demonstrated dose-dependent binding of eRNA to wild-type (wt) Eno (Fig. 5A). Substitution of lysine 434 with glycine (Eno^K434G^) markedly reduced the eRNA binding ability of Eno (Fig. 5A). Efficient binding of eDNA to wt Eno and a marked reduction in Eno binding ability of K434G mutant were observed as well (Fig. 5A). As binding of Plg to Eno strongly depends on K434, Plg was used as a positive control (Fig. 5A). To confirm results obtained by the slot-blot filter binding assay, a solid-phase binding assay was performed (Fig. 5B–D). Here, a microtiter plate was coated with wt Eno, Eno^K434G^ or BSA and increasing concentrations of nucleic acids were applied. While the efficient binding of eRNA and eDNA to wt Eno was observed, the interaction of Eno^K434G^ with nucleic acids was severely impaired (Fig. 5B,C). As expected, the K434 substitution with glycine abolished also the Plg/Eno interaction (Fig. 5D).

Taken together, our data provide the evidence that C-terminal lysine residue 434 of Eno is important for its interaction with extracellular nucleic acids.

### Extracellular RNA-dependent S. pneumoniae invasion is not affected by the deletion of C-terminal lysine residues and the mutation of Eno internal Plg-binding motif

**Figure 6:**
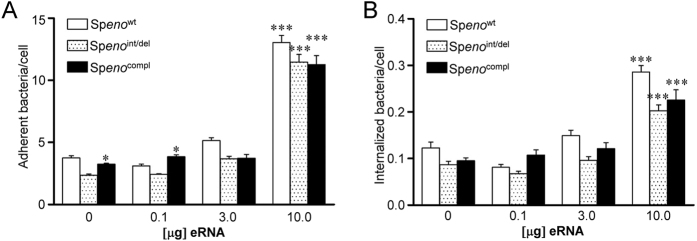
Pneumococcal Eno mutants lacking C-terminal eRNA-binding motif do not demonstrate reduced adherence to and internalization into A549 cells. (**A,B**) A549 cells were preincubated with different amounts of eRNA and infected with *S. pneumoniae* overexpressing wt Eno (Sp*eno*^wt^) or lacking C-terminal eRNA-binding motif (Sp*eno*^int/del^). In rescue experiments Sp*eno*^int/del^ was preincubated with soluble Eno protein (Sp*eno*^compl^). Pneumococcal host-cell adherence (**A**) and internalization (**B**) were determined by immunofluorescence staining and microscopy. Data represent mean values ± SEM; n = 3; *p ≤ 0.05 *vs* Sp*eno*^int/del^; ***p ≤ 0.001 *vs* untreated A549 cells.

To study the importance of eRNA-binding motifs within Eno on the eRNA-triggered bacterial invasion, a *S. pneumoniae* strain carrying deletion of the C-terminal lysines (^433^KK^434^) and amino acid substitutions in the internal Plg-binding motif (K251L, E252G and K254L) of Eno (Sp*eno*^int/del^) was employed^19^. As a control, *S. pneumoniae* overexpressing wt Eno (Sp*en*o^wt^) was utilized^19^. A549 cells were infected with both bacterial strains and their adherence to A549 cells and internalization was quantified (Fig. 6A and B, respectively). Sp*eno*^wt^ strain demonstrated increased adherence to and internalization into A549 cells in the presence of 10 μg of eRNA (Fig. 6A,B). However, the eRNA-mediated adherence of Sp*eno*^int/del^ to A549 cells was only slightly diminished as compared to the Sp*eno*^wt^ strain (Fig. 6A). In addition, only a moderate tendency towards reduction in internalization of Sp*eno*^int/del^ into A549 cells as compared to Sp*eno*^wt^ strain was noted (Fig. 6B). As reassociation of soluble Eno protein to bacterial surface was previously reported^17^, bacteria were preincubated with soluble Eno protein in order to restore Eno-dependent binding capacity of Sp*eno*^int/del^ (Fig. 6A,B). Only, in the absence of eRNA or in the presence of 0.1 μg eRNA, pretreatment of Sp*eno*^int/del^ with Eno protein (Sp*eno*^compl^) resulted in increased bacterial adherence (Fig. 6A). No significant differences in Sp*eno*^compl^ internalization into A549 cells as compared to Sp*eno*^int/del^ were noted (Fig. 6B).

In summary, the deletion of C-terminal lysine residues and the mutation of internal Plg-binding motif only marginally impaired *S. pneumoniae* adherence and internalization pointing to the role of other Eno eRNA-binding sites in bacteria invasion or the existence of additional eRNA-binding proteins on *S. pneumoniae* surface.

### Extracellular eRNA-binding proteins are expressed on *S. pneumoniae* surface

**Figure 7:**
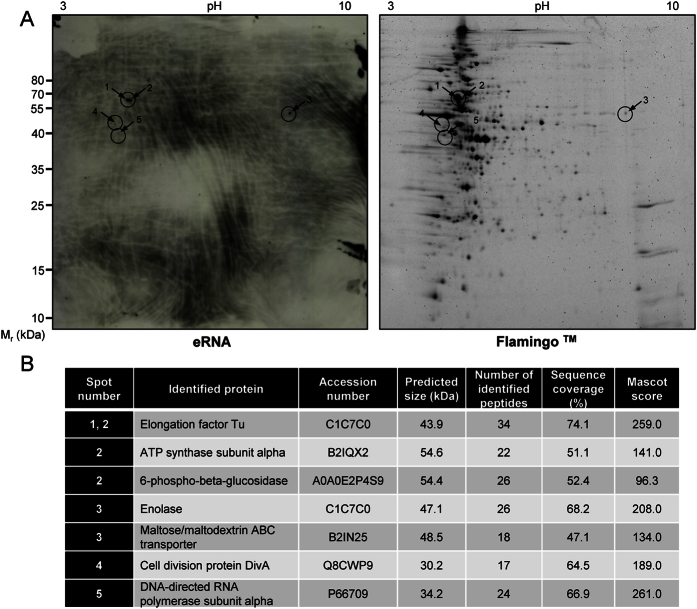
Identification of eRNA-binding proteins associated with pneumococcal wall. (**A**) Two hundred fifty μg of cell wall proteins isolated from *S. pneumoniae* serotype 2 strain (ATCC11733), were resolved using two-dimensional gel electrophoresis. Polyacrylamide gels were simultaneously subjected to Western blotting using biotinylated eRNA (left panel) and to flamingo fluorescent gel stain (right panel). (**B**) List of proteins identified by MALDI-TOF analysis.

To identify other cell wall-associated proteins that interact with eRNA, protein wall fraction of *S. pneumoniae* was isolated and subjected to proteomic analysis (Fig. 7A). Cell wall proteins were separated using 2-D gel electrophoresis and eRNA-bound proteins were identified by MALDI-TOF MS. This approach revealed seven eRNA-binding proteins on the pneumococcal surface including: elongation factor Tu (EFTu; spots 1 and 2), ATP synthase subunit alpha (ATPα; spot 2), 6-phospho-beta-glucosidase (BglA3; spot 2), maltose/maltodextrin ABC transporter (MalABC; spot 3), Eno (spot 3), cell division protein DivA (spot 4), DNA-directed RNA polymerase subunit alpha (RNAPα, spot 5) (Fig. 7B). Interestingly, Eno exhibited a moderate eRNA binding capacity as compared to EFTu, ATPα and BglA3, which manifested more pronounced interactions with eRNA (Fig. 7A, left panel).

### RNase1 treatment inhibits eRNA-triggered *S. pneumoniae* invasion

**Figure 8:**
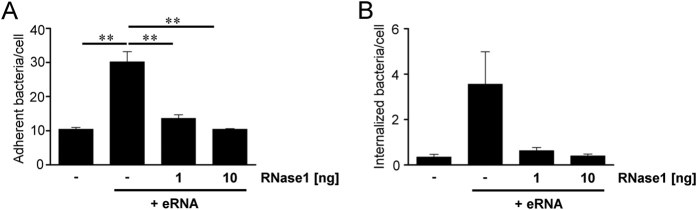
Extracellular RNA-mediated pneumococcal infection is diminished after RNase1 treatment. (**A,B**) A549 cells were preincubated with eRNA (5 μg) and infected with Sp*Δply* pneumococci in the absence or presence of RNase1 (1 and 10 ng). Pneumococcal host-cell adherence to (**A**) and internalization (**B**) into A549 cells were tested using antibiotic protection assay. Data represent mean values ± SEM; n = 3; **p ≤ 0.01.

In order to confirm the eRNA-mediated effect on pneumococcal adherence and invasion, eRNA-preincubated lung epithelial cells were treated with 1 ng or 10 ng RNase1 prior to the infection with Sp*Δply* pneumococci. A three-fold increase in bacterial adherence was determined after preincubation of A549 cells with 5 μg of eRNA resulting in a relative amount of 30.2 ± 2.2 bacteria per cell *versus* only 10.4 ± 0.6 attached bacteria per cell without eRNA (Fig. 8A). Treatment of A549 cells with 1 ng RNase1 reduced eRNA-mediated pneumococcal attachment down to 13.6 ± 1.2 adherent bacteria per cell (Fig. 8A). Incubation of eRNA-treated A549 cells with 10 ng RNAse1 completely neutralized the supporting effect of the eRNA on bacterial adherence. Similar results were obtained for the amount of internalized bacteria, although internalization rates in general were below 1 bacteria per cell (Fig. 8B). In order to quantify the bacterial internalization, the amount of internalized bacteria per 2 × 10^5^ cells was analyzed by antibiotic protection analyses. The counted cfu of invaded pneumococci revealed an up to 10-fold higher internalization rate after eRNA preincubation as compared to eRNA-free A549 cells (Fig. 8B). Incubation of A549 cells with 1 and 10 ng RNase1 completely abolished promoting effect of eRNA on *S. pneumoniae* internalization (Fig. 8B). No impact of RNase1 on A549 viability was observed (data not shown). These results imply that RNase1 may markedly diminish eRNA-mediated invasion of *S. pneumoniae* and confirm the role of eRNA as cofactor in pneumococcal adherence and internalization.

## Discussion

In the current study we described the impact of extracellular host-derived RNA on the pneumococcal pathogenicity. Here, we provide compelling evidence that eRNA significantly promotes *S. pneumoniae* adhesion to and internalization into different host cells including HUVEC, HPMEC and A549 cells. The mechanism involves direct interaction of eRNA with bacterial cell wall-associated proteins to serve as a bridging factor between bacteria and host cells. Using a mass spectrometry approach novel eRNA-binding proteins potentially involved in eRNA-mediated pneumococcal invasion were identified. We demonstrated, for the first time, that Eno, a well-characterized Plg-receptor, strongly interacts with eRNA and identified multiple eRNA-binding sites: ^59^RYGGLGTQK^67^, ^104^KGKLGA^109^, ^188^HALKKILKSRGLETA^202^, ^312^GKKVQL^317^ and ^432^LKK^434^ within the Eno sequence. Interestingly, ^432^LKK^434^ motif is involved in interaction with Plg as well^17^,^18^. Although, substitution of lysine 434 with glycine (K434G) markedly diminished the binding of eRNA to Eno, the adherence to and internalization into alveolar epithelial cells of *S. pneumoniae* strain carrying the C-terminal lysine deletion and the mutation of internal Plg-binding motif were not significantly impaired. Consequently, proteomic analysis of cell wall fraction of *S. pneumoniae* identified, in addition to Eno, several surface-associated eRNA-binding proteins. These include: EFTu, ATPα, BglA3, MalABC, DivA, and RNAPα. Interestingly, treatment with RNase1 completely inhibited eRNA-mediated pneumococcal adherence to and internalization into alveolar epithelial cells. Together, these data indicate that eRNA represents a novel virulence cofactor promoting pneumococcal invasion and that treatment with RNase1 may provide a novel therapeutic option for patients with pulmonary infectious diseases.

Adhesion of bacteria to host tissue is an essential step in microbial colonization and the development of complex prokaryotic communities. It is also a prerequisite for the majority of infectious diseases^6^. Bacteria utilize adhesins to bind to eukaryotic cell surface receptors and ECM^6^, which is composed of proteoglycans as well as fibrous (among others fibronectin, collagen, elastins and laminins) and matricellular (vitronectin and TSP-1) proteins^33^,^34^,^35^. Immobilized laminin, fibronectin and collagens were shown to interact with the bacterial proteins such as pneumococcal adherence and virulence factor B (PavB) and the tip of the pneumococcal pilus-1 structure (RrgA), leading to effective positioning of the bacteria and invasion of adjacent epithelial cells^6^. It was also demonstrated that human TSP-1 is recruited to the *S. pneumoniae* surface *via* bacterial peptidoglycan, and it can be exploited as a molecular bridge between bacterium and host cell^9^,^36^. Moreover, pneumococci engage ECM vitronectin to adhere to host ανβ3 integrins, promoting bacterial internalization^37^. In the current study, we propose that host-derived nucleic acids, in particular eRNA, which are released from epithelial cells in response to infectious mediators, may potentiate *S. pneumoniae* colonization by increasing bacterial invasive properties. The mechanism involves the binding of eRNA to pneumococcal cell wall components, among which Eno was previously identified as a nucleic acid-binding protein^38^. Our data revealed that the binding of the eRNA to the EnoK434G mutant was markedly attenuated as compared to wt Eno. This suggests that eRNA may share the C-terminal binding motif with Plg, most likely without affecting Eno-mediated recruitment of Plg of *S. pneumoniae*, since Eno may also interact with Plg by internal Plg-binding region (^248^FYDKERKVY^256^)^39^. Interestingly, results of our protein binding assays demonstrate that the pneumococcal Eno binds not only eRNA, but also eDNA. However, incubation of eukaryotic cells with eDNA had no significant effect on bacterial adherence. This result may be explained by the presence of DNA-specific ribonucleases on bacterial surface. Pneumococci express eDNA-cleaving endonucleases to promote bacterial escape from neutrophil extracellular traps^40^. Thus, the eDNA-specific nuclease activity may neutralize the effect of eDNA on bacterial invasion.

Surprisingly, the deletion of the C-terminal lysine residues and the mutation of the internal Plg-binding region had almost no impact on eRNA-driven bacterial adhesion and internalization. This indicates that either other eRNA-binding motifs of Eno are involved in eRNA-triggered pneumococcal invasion or bacterial cells surface exposes other eRNA-binding proteins, which effectively compensate for the lack of Eno. Our proteomic analysis revealed seven potent eRNA-binding proteins. Interestingly, some of these proteins share similarities with bacterial Eno. For instance, RNAPα and BglA3 possess C-terminal lysine residues. Although C-terminal lysine residue is absent in EFTu, this protein has been identified as a Plg receptor^41^,^42^,^43^. Instead of C-terminal lysines, it seems that EFTu/Plg interaction occur *via* internal amino acid regions (^179^LKALEGDSHYEDIV^192^ and ^249^VGIKEETQKAV^259^). Prediction analysis of these fragments revealed that the amino acid charge meets the requirements for the interaction with lysine-binding sites of the Plg-kringle domains (S. Bergmann, unpublished data). Apart from Plg binding, cytoplasm-localized EFTu regulates protein synthesis and cellular metabolism by binding to and transporting amino-acyl-tRNA to the ribosomes confirming its RNA-binding ability^44^. Since EFTu, similarly to Eno, is associated with the cell wall of various bacteria^41^,^42^,^43^, it is imaginable that other cytosolic proteins such as RNAPα and/or BglA3 may also be transported to the bacterial surface thereby acting as “moonlighting proteins” for eRNA-binding at certain steps of the growth phase.

As a consequence of pneumococcal infection followed by cellular disruption sustained during acute lung injury, nucleic acids may be liberated to the lung parenchyma^20^,^45^,^46^. Increased level of eRNA was demonstrated in bronchoalveolar lavage fluids of patients suffering from acute respiratory distress syndrome (ARDS)^47^. This eRNA may participate in ARDS pathogenesis by increasing permeability of epithelia-capillary barrier^48^, enhancing intraalveolar fibrin deposition^22^,^23^,^47^,^49^ and stimulating the production and release of proinflammatory cytokines^22^,^23^,^26^,^27^,^49^. Here, we propose a novel function of eRNA, which promotes *S. pneumoniae* infection and the development of pneumonia, a well-described direct cause of ARDS^50^. Given the relative high number of eRNA-binding proteins on pneumococcal surfaces, the best strategy to reduce eRNA-driven infection is to eliminate the excess of eRNA, rather than target one specific eRNA-binding protein. RNase is a natural counterpart of RNA in the vascular system^20^. Several RNases possess diverse types of antimicrobial properties. However, it is unclear whether the killing of pathogens requires their intact enzymatic activity. From one hand, it was demonstrated that inhibition of ribonuclease activity strongly suppressed antifungal properties of RNase5 and RNase7^51^, but on the other hand, studies using a ribonuclease-inactive recombinant RNase7 revealed that RNase7 activity is not essential for killing Gram-negative and Gram-positive bacteria including *P. aeruginosa* and *S. pneumoniae*^52^,^53^,^54^. The ability to bind to bacterial surface and to permeabilize the bacterial membrane seems to be crucial in antimicrobial action of many RNases^52^,^53^. This mechanism, however, does not apply to RNase1 activity against *S. pneumoniae*, since our results did not show any influence of RNase1 on bacterial survival. Thus, it is imaginable that the RNase1-mediated degradation of eRNA, a new adhesin, is partially responsible for anti-*pneumococcal* properties of this ribonuclease. Regardless of the mechanism, our *in vitro* data demonstrate that RNase1 treatment leads to the significant inhibition of *S. pneumoniae* adhesion to and internalization into alveolar epithelial cells and suggest that RNase1 may prevent the bacterial colonization in the lung. RNase1 has been successfully used as effective tissue-protective regimen in several animal models of human diseases. For instance, the administration of the RNase1 significantly reduced thrombus formation, prevented stroke and diminished the development of brain oedema^48^. Furthermore, RNase1 inhibited neointima development in an atherosclerotic mouse model^55^, and, in addition, conferred cardiac protection against ischaemia/reperfusion^26^,^56^,^57^. In subcutaneous xenograft models of human cancer, RNase1 strongly counteracted the tumorigenic activities of eRNA^25^. However, further studies utilizing murine model of pneumococcal infection are needed to validate whether the administration of RNase1 may prevent the development of *S. pneumoniae*-driven infection diseases.

In summary, we demonstrate for the first time that eRNA potentiates *S. pneumoniae* infection of alveolar epithelial cells. Since treatment with RNase1 inhibited eRNA-triggered *S. pneumoniae* invasion, our data support further efforts to employ RNAse1 as an antimicrobial agent to combat pneumococcal infectious diseases.

## Methods

### Materials

The His6-tagged *S. pneumoniae* Eno and Eno^K434G^ were expressed in the *Escherichia coli* host strain M15 (pREP4) and protein purification was performed as previously described^17^. Lys-Plg was purchased from Chromogenix (Mölndal, Sweden), tranexamic acid (TXA) from Sigma-Aldrich (Taufkirchen, Germany) and t-PA from American Diagnostica (Pfungstadt, Germany). Recombinant bovine RNase1 and serum albumin (BSA) were purchased from Fermentas/Thermo Scientific (Schwerte, Germany) and from Sigma Aldrich, respectively.

### *Streptococcus* strains and media

*Streptococcus pneumoniae* strains were cultured in Todd-Hewitt broth (Oxoid, Basingstoke Hampshire, UK) supplemented with 0.5% yeast extract (THY) to mid log phase or were cultured on Colombia blood agar plates containing 5% sheep blood (Becton Dickinson, Heidelberg, Germany). A low encapsulated serotype 2 pneumococcus (ATCC 11733) and isogenic Eno mutants were employed in cell culture infection experiments^17^. Pneumococcal mutants expressing Eno, lacking Plg-binding sites, were generated *via* site-specific deletion-insertion mutagenesis using the genetic background of the serotype 2 strain (ATCC 11733) carrying the gene for erythromycin resistance^17^. The strain Sp*eno*^int/del^ carries a deletion of the C-terminal lysines (^433^KK^434^) and amino acid substitutions in the internal Plg-binding motif (K251L, E252G and K254L) of Eno^19^. The isogenic mutant Sp*eno*^wt^ overexpresses wt Eno and was used as control. A pneumolysin-deficient serotype 35 A/47 mutant strain (Sp*Δply*) was employed in some cell culture infection analysis to exclude pneumolysin-dependent effects on cell viability during assay conduction. The mutants were cultured on media supplemented with 5.0 μg/ml erythromycin.

### Cell culture

Human lung alveolar epithelial cells type II pneumocytes (A549 cells, ATCC CCL-185) were cultured in Dulbecco’s modified Eagle’s medium (DMEM) with low glucose (Invitrogen Life Technologies, Carlsbad, CA), supplemented with 10% (v/v) heat-inactivated fetal bovine serum (FBS; Hyclone, Cramlington, UK) and 2 mM L-glutamine (Invitrogen Life Technologies) at 37 °C and 5% CO_2_.

### Binding of eRNA to enolase peptides synthesized on a cellulose membrane

A membrane with 141 spot-synthetized peptides, derived from the Eno amino acid sequence, consisting of 15 amino acids each with an offset of three amino acids, was prepared as previously described^19^. eRNA binding was performed according to a standardized protocol as previously reported^58^. In brief, the membrane was incubated for 7 h with blocking buffer (Genosys Biotechnologies, London, UK) followed by overnight incubation with 5 μg biotinylated eRNA diluted in blocking buffer. After washing, the membrane was incubated for 2 h with peroxidase-conjugated Streptavidin (Dako, Glostrup, Denmark). Finally, eRNA binding to peptides was detected using Chemiluminescence Reagent Plus substrate (Thermo Scientific) and a Kodak Image Station 2000 R. Quantitative densitometry analysis was performed using 1D gel analysis software LabImage 1D (Intas, Goettingen, Germany).

### Binding of RNA, DNA and plasminogen to pneumococcal enolase

The extraction of RNA from A549 cells was performed using QIAzolTM lysis reagent (Qiagen, Hilden, Germany) according to the manufacturer’s instructions. Genomic DNA was isolated from A549 cells using Genomic DNA Kit (Qiagen) following the manufacturer’s instructions. For fragmentation of nucleic acids, 1 mg of biotinylated RNA or DNA was dissolved in 1 ml of RNase-, DNase-free water and sonicated for 2, 4, 6, 8, 10, or 12 s. At the indicated time points, 100 μl samples were withdrawn, and nucleic acids were precipitated with ethanol. The RNA and DNA were dissolved in RNase- and DNase-free water and tested for Eno-binding capacity. Parallel samples were subjected to agarose gel electrophoresis and visualized by ethidium bromide staining.

The nucleic acid/Eno-binding activity was assessed by a filter-binding assay, where a nylon membrane was soaked with buffer A [10 mM Tris-Cl (pH 7.5), 50 mM NaCl, 1 mM EDTA, and 1× Denhardt’s solution (0.02% (m/v) Ficoll, 0.02% (m/v) polyvinylpyrrolidone, and 0.02% (m/v) BSA)] and fixed into a slot-blot apparatus^47^. Increasing concentrations (0–6 μg/ml) of wild-type Eno (Eno^wt^), Eno variant (Eno^K434G^) or BSA were applied to the filter in 100 μl of Tris-buffered saline (TBS) for 15 min, and then aspirated through the filter and cross-linked for 10 min by exposure to ultraviolet light (254 nm). After washing with buffer A, the filter was incubated with either 10 μg/ml of biotinylated eRNA, 3 μg/ml of biotinylated eDNA or 10 μg/ml Plg. Subsequently, the filter was washed and finally incubated with peroxidase-labeled streptavidin (DAKO). Detection of eRNA, eDNA and Plg was accomplished using enhanced chemiluminescence (Thermo Scientific). Biotinylation of RNA and DNA was performed with EZ LinkTM Psoralen-PEO-biotin (Thermo Scientific) according to the manufacturer’s instructions. Plg was biotinylated using the EZ-Link^TM^ Sulfo-NHS-Biotinylation Kit (Thermo Scientific)^59^.

A microtiter plate was coated with Eno, Eno variant (Eno^K434G^) or BSA (6 μg/ml each) in 50 nM NaHCO_3_, pH 9.6, and incubated at 4 °C overnight. The plate was washed three times with Tris-buffered saline (TBS; 150 mM NaCl, 20 mM Tris, pH 7.5) containing 0.1% Tween (TBS-T) buffer, and nonspecific binding sites were blocked with 3% (m/v) BSA in TBS-T at room temperature for 1 h. Increasing concentrations of biotinylated Plg (0–25 μg/ml), eRNA (0–25 μg/ml) or eDNA (0–1 μg/ml) in TBS were added to the wells and biotinylated molecules were allowed to bind to the Eno^wt^, Eno^K434G^ or BSA at room temperature for 2 h. After extensive washing with TBS, bound Plg, eRNA or eDNA were detected using a peroxidase-labeled streptavidin. Final detection was performed using 3,3′,5,5′ tetramethylbenzidine (TMB), with a TMB substrate kit (Thermo Scientific) according to the manufacturer’s instructions.

### Infection experiments and quantification of pneumococcal adherence and internalization into host cells by immunofluorescence staining and microscopy

eRNA-mediated bacterial adherence was quantified by counting the amount of bacteria attached to host cells after differential immunofluorescence staining of the pneumococci as previously described^29^. The impact of Eno/eRNA interaction on pneumococcal adherence was studied using a serotype 2 *S. pneumoniae* strain (ATCC11733) and isogenic enolase mutants^17^. Quantification of internalized bacteria was performed by antibiotic protection assay as previously described^29^. For details, please refer to the supplement.

### Fractionation of pneumococcal cell wall proteins

Cell wall proteins from a serotype 2 *S. pneumoniae* strain (ATCC1173) were isolated by sequential fractionation as previously described^17^. For details, please refer to the supplement.

### Determination of net peptide charge

Protein Calculator v3.4, developed by Chris Putnam at the Scripps Research Institute (www.scripps.edu/~cdputnam/protcalc), was used to calculate the net charge at physiological pH (7.4) for different peptide sequences.

### Identification of eRNA binding proteins in *S. pneumoniae* cell wall fraction

For two-dimensional (2-D) gel electrophoresis 250 μg of cell wall proteins isolated from *S. pneumoniae*, a serotype 2 strain (ATCC11733), were used. Protein separation in the second dimension was performed by electrophoresis on 12.5% SDS polyacrylamide gels. Electrophoresis was carried out in a Hoefer 600 system. The matrix-assisted laser desorption/ionization mass spectrometry-time of light mass spectrometry (MALDI-TOF-MS) was performed on an Ultraflex TOF/TOF mass spectrometer equipped with a nitrogen laser and a LIFT-MS/MS facility. For details, please refer to the supplement.

### Flow cytometry

A549 cells were seeded into 6-well tissue culture plates at a density of 2 × 10^5^ cells/well. Next day, cells were wasched with PBS and incubated with 0.1, 1, 5 and 10 μg of biotinylated or unlabeled eRNA in 1 ml of DMEM medium for 1 h at 37 °C. Afterwards, the cells were washed twice with PBS and incubated with Cy^®^5-conjugated Streptavidin (Invitrogen Life Technologies) for 1 h at 37 °C. After additional washing steps, cells were collected by scratching and the binding of eRNA to cell membrane was analyzed in the Cy^®^5 channel of the flow cytometer. Data were collected using an Accuri C6 flow cytometer (BD Biosciences, Heidelberg, Germany) and analyzed by CFlowPlus (BD Biosciences). Gates based on forward and side scatter were set to eliminate cellular debris and cell clusters. A minimum of 2 × 10^4^ cells were analyzed per sample. As an additional control, cells incubated with unlabeled eRNA and incubated with Cy^®^5-conjugated streptavidin were used.

### Statistics

The statistical analysis was performed using a GraphPad Prism version 5.02 for Windows (GraphPad Software, La Jolla, CA). Differences between groups were assessed using the Student’s *t*-test. When three or more groups were compared, analysis of variance (ANOVA) followed by Tukey’s *post hoc* test was used. Experimental data are presented as mean (±SEM). p value less than 0.05 was considered statistically significant.

## Additional Information

**How to cite this article**: Zakrzewicz, D. *et al*. Host-derived extracellular RNA promotes adhesion of *Streptococcus pneumoniae* to endothelial and epithelial cells. *Sci. Rep.* **6**, 37758; doi: 10.1038/srep37758 (2016).

**Publisher's note:** Springer Nature remains neutral with regard to jurisdictional claims in published maps and institutional affiliations.

## Supplementary Material

Supplementary Information
