# Supplementary material for: Host-derived extracellular RNA promotes adhesion of Streptococcus pneumoniae to endothelial and epithelial cells
Source: Sci Rep. 2016 Nov 28;6:37758. doi: 10.1038/srep37758 (PMC5125276; doi:10.1038/srep37758)
Supplement: Supplementary Information [file srep37758-s1.pdf]

ONLINE SUPPLEMENTAL MATERIAL

Host-derived extracellular RNA promotes adhesion of *Streptococcus pneumoniae* to endothelial and epithelial cells

Dariusz Zakrzewicz[1,*,†], Simone Bergmann[2,7,†], Miroslava Didiasova[1], Benedetto Daniele Giaimo[1], Tilman Borggrefe[1], Maren Mieth[3], Andreas C. Hocke[3], Guenter Lochnit[1], Liliana Schaefer[4], Sven Hammerschmidt[5], Klaus T. Preissner[1] and Malgorzata Wygrecka[1,6]

[1]Department of Biochemistry, Faculty of Medicine, Universities of Giessen and Marburg Lung Center, Friedrichstrasse 24, 35392 Giessen, Germany;
[2]Department of Medical Microbiology, Helmholtz Centre for Infection Research, Inhoffenstrasse 7, 38124 Braunschweig, Germany;
[3]Department of Internal Medicine/Infectious Diseases and Pulmonary Medicine, Charité-University Medicine Berlin, Chariteplatz 1, 10117 Berlin, Germany;
[4]Institute of Pharmacology and Toxicology, Goethe University School of Medicine, University Hospital, Theodor Stern Kai 7, 60590 Frankfurt am Main, Germany
[5]Department Genetics of Microorganisms, Interfaculty Institute for Genetics and Functional Genomics, Ernst-Moritz-Arndt Universität Greifswald, Friedrich-Ludwig-Jahn-Strasse 15a, 17489 Greifswald, Germany;
[6]Member of the German Center for Lung Research, Giessen

Present address: [7]Department of Infection Biology, Institute of Microbiology, Technische Universität Braunschweig, Biocentre, Spielmannstrasse 7, 38106 Braunschweig, Germany

[*]To whom correspondence should be addressed: Dariusz Zakrzewicz, PhD, Department of Biochemistry, Faculty of Medicine, Universities of Giessen and Marburg Lung Center, Friedrichstrasse 24, 35392 Giessen, Tel.: +49 641 9947508, Fax: +49 641 9947509,
E-mail: dariusz.zakrzewicz@innere.med.uni-giessen.de

[†]These authors equally contributed to this work

METHODS

Cell culture

Human umbilical vein endothelial cells (HUVEC) and human pulmonary microvascular endothelial cells (HPMEC) were purchased from Promocell and cultured in Endothelial Cell growth Medium MV (ECGM MV, Promocell) with the specific ECGM MV-supplement kit (Promocell) at 37 °C and 5 % CO_2. The primary endothelial cells were used in very early passages only.

Infection experiments and quantification of pneumococcal adherence and internalization into host cells by immunofluorescence staining and microscopy

Bacterial adherence was quantified by counting the amount of bacteria attached to host cells after differential immunofluorescence staining of the pneumococci as previously described[1]. In brief, the eukaryotic cells were seeded on glass cover slips (diameter 12 mm) and cultivated to sub-confluence (~2×10^5 eukaryotic cells). In order to analyze the adherence and internalization of eRNA-treated pneumococci, the eukaryotic cells were incubated with eRNA isolated from A549 (0.01 µg – 10 µg) for 30 min or with 5 µg eRNA and 1 ng RNAse1 or 10 ng RNAse1 alone at 37°C and 5.0 % CO_2. After removal of unbound RNA via PBS-washing, host cells were incubated for 4 h with pneumococci using a MOI of 50 per host cell for 4 h at 37 °C and 5.0% CO_2 in infection medium (DMEM with low glucose supplemented with 1 % FBS). Thereafter, unbound bacteria were removed by rinsing the wells three times with infection medium. The effective infection dose (colony forming units, CFU) per well was determined by serial plating of bacteria on blood agar plates. Bacterial adherence was quantified by counting the amount of bacteria attached to host cells after differential immunofluorescence staining of the pneumococci

as described earlier[1]. Prior to microscopic quantification, the infected host cells were fixed on the glass coverslips with 3.0 % PFA in PBS. The staining procedure resulted in Alexa568-labeled intracellular bacteria (red fluorescence) and Alexa488/568-labeled extracellular pneumococci (green/yellow). The number of attached and intracellular bacteria was counted by immunofluorescence microscopy using at least 60 eukaryotic cells. Image acquisition was done with a confocal laser scanning microscope (Leica TCS SP5 AOBS) and the LAS AF SP5 software. The impact of Eno-PLG interaction on pneumococcal adherence was studied using a serotype 2 *S. pneumoniae* strain (ATCC11733) and isogenic enolase mutants. Quantification of internalized bacteria was performed by antibiotic protection assay as described in [1]. In brief, to quantify the number of internalized and recovered viable pneumococci, eRNA-pretreated A549 cells were incubated with pneumococci at an MOI of 50 for 3 h followed by one hour incubation with infection medium containing 100 U/ml penicillin and 0.1 mg/ml gentamycin at 37 °C. After six washing steps with the infection medium, the eukaryotic cells were lysed for 10 min at 37 °C with 1 % (w/v) saponine solution in PBS. Thereafter, the cell suspensions were plated in serial dilutions on blood agar plates and after overnight culture at 37 °C and 5.0 % CO_2 the internalized and recovered bacteria were enumbered by counting the cfu. All infection analyses were performed at least three times in triplicates.

Fractionation of pneumococcal cell wall proteins

Fractionation of pneumococcal cell wall proteins was performed as previously described[2]. Briefly, bacteria were grown to mid-log phase, harvested and washed twice with 0.1 M Tris-HCl buffer (pH 7.2). The bacterial sediment was resuspended in 10 ml of Tris-HCl buffer supplemented with 1 mM phenylmethylsulfonid fluorid (PMSF; Sigma aldrich) on ice.

Mechanical cell lysis was carried out by two passages through a French press. Intact bacteria were removed by centrifugation for 15 min at 3000 g. The cell wall fraction was separated from the cytosolic fraction by centrifugation for 50 min at 19 000 g. The cell wall fraction was resuspended in 0.1 M potassium phosphate buffer (pH 7.2) containing 150 mM KCl, 10 mM EDTA and 20 % glycerol. Solubilisation of the cell wall proteins was performed by sequential treatment for 30 min at 30 °C in the presence of 5 mM, 10 mM, 20 mM and 50 mM detergent CHAPS 3-[(3-cholamidopropyl)- dimethylammonio]-1-propanesulphonate. After centrifugation for 30 min at 19 000 g, the supernatants were collected, combined and precipitated with ammonium sulphate. Finally, probes were dialysed in PBS overnight at 4 °C.

Identification of eRNA binding proteins in *S. pneumoniae* cell wall fraction

For two-dimensional (2-D) gel electrophoresis 250 μg of cell wall proteins isolated from *S. pneumoniae*, a serotype 2 strain (ATCC11733), were solubilized in 6 M urea, 2 M thiourea, 4 % CHAPS, 1 % DTT and 2 % Pharmalyte 3-10. IPG-strips (pH 3-10 NL) were rehydrated at 20 °C with the protein extract. On each strip, 250 μg proteins were applied and isoelectric focusing was performed with 32.05 kVh. After focusing, the IPG-strips were equilibrated for 10 min in 2 ml equilibration stock solution (ESS; 6 M urea, 0.1 mM EDTA, 0.01 % bromphenol blue, 50 mM Tris-HCl pH 6.8, 30 % glycerol) for 15 min in 2 ml ESS I (10 ml ESS containing 200 mg SDS, 100 mg DTT) followed by 15 min in ESS II (10 ml ESS containing 200 mg SDS, 480 mg iodacetamide). Protein separation in the second dimension was performed by electrophoresis on 12.5 % SDS polyacrylamide gels. Electrophoresis was carried out in a Hoefer 600 system with the following program: 15 min at 15 mA/gel and 5 h at 110 mA at 25 °C. Gels were stained with Flamingo (Bio-rad Laboratories GmbH, Munich, Germany) and scanned with a Typhoon 9100

(GE Healthcare, Munich, Germany). Selected spots were digested after reduction and carbamidomethylation with trypsin. Tryptic peptides were eluted from the gel plugs with 1 % trifluoric acid. The matrix-assisted laser desorption/ionization mass spectrometry-time of light mass spectrometry (MALDI-TOF-MS) was performed on an Ultraflex TOF/TOF mass spectrometer equipped with a nitrogen laser and a LIFT-MS/MS facility. The instrument was operated in the positive-ion reflectron mode using 2.5-dihydroxybenzoic acid and methylendiphosphonic acid as matrix. Sum spectra consisting of 200 – 400 single spectra were acquired. For data processing and instrument control the Compass 1.1 software package consisting of FlexControl 2.4, FlexAnalysis 3.0 and BioTools 3.0 was used. Proteins were identified by MASCOT peptide mass fingerprint search using the Uniprot Bacteria database (20150624; 11624423 sequences; 3649040132 residues). For the search a mass tolerance of 75 ppm was allowed and carbamidomethylation of cysteine as global modification and oxidation of methionine as variable modification were used. A false positive rate of 5% was allowed.

Determination of bacterial growth in infection medium

S. pneumoniae ATCC11733 was grown in THY medium to mid log phase, sedimented by centrifugation and adjusted to 1 x 10^7 bacteria/ml in infection medium (DMEM + 1 % FBS). Bacteria were cultivated at 37 °C and 5% CO_2. Growth of the bacteria was determined by photometrical measurement at 600 nm in time intervals of 30 min for up to 4h. Measurement was performed in triplicates using THY or infection medium for blank subtraction. The following incubation set ups were analyzed: THY medium, cell culture infection medium (DMEM + 1% FBS), infection medium with 1 µg/ml eRNA, infection medium with 10 µg/ml eRNA, and infection medium with 10 ng RNase1.

FIGURE LEGENDS

Figure S1. Pneumococcal infection is enhanced in presence of eRNA. (A, B) HUVEC (A) and HPMEC (B) were preincubated with the eRNA (5 µg) and pneumococcal host-cell adherence (left panel) and internalization (right panel) of serotype 2 *S. pneumoniae* (Sp)-strain (ATCC11733) were measured by immunofluorescence staining and microscopy. Data represent mean values ± SEM; n=3; *, $p \leq 0.05$ *vs* untreated A549 cells.

Figure S2. Replication of *S. pneumoniae* ATCC11733 in THY medium and in cell culture infection medium supplemented with eRNA or RNAse1. Bacteria were prepared as described for infection analyses and incubated for up to 4 h in THY medium (●), infection medium (DMEM + 1%FBS) (◊), infection medium with 1 µg/ml eRNA (Δ), infection medium with 10 µg eRNA (□), and infection medium with 10 ng RNase (○). The growth of bacteria was monitored by measuring optical density at 600 nm.

Figure S3. Extracellular RNA binds to Gram-positive and Gram-negative bacteria. Gram-positive (*Streptococcus pneumoniae 35*, *Streptococcus pneumoniae 35A Δpl, Streptococcus pneumoniae TIGR4, Streptococcus pneumoniae TIGR4 ΔendA, Staphylococcus aureus, Streptococcus pyogenes* and *Listeria monocytogenes)* and Gram-negative (*Neisseria meningitodis* and *Escherichia Coli)* bacteria were immobilized on a nitrocellulose membrane. The membrane was incubated with biotinylated eRNA and the binding of eRNA to bacteria was evaluated by chemiluminescence.

REFERENCES

1. Bergmann, S., Schoenen, H. & Hammerschmidt, S. The interaction between bacterial enolase and plasminogen promotes adherence of Streptococcus pneumoniae to epithelial and endothelial cells. *Int J Med Microbiol* **303**, 452-462 (2013).
2. Bergmann, S., Rohde, M., Chhatwal, G. S. & Hammerschmidt, S. alpha-Enolase of Streptococcus pneumoniae is a plasmin(ogen)-binding protein displayed on the bacterial cell surface. *Molecular microbiology* **40**, 1273-1287 (2001).

FIGURES

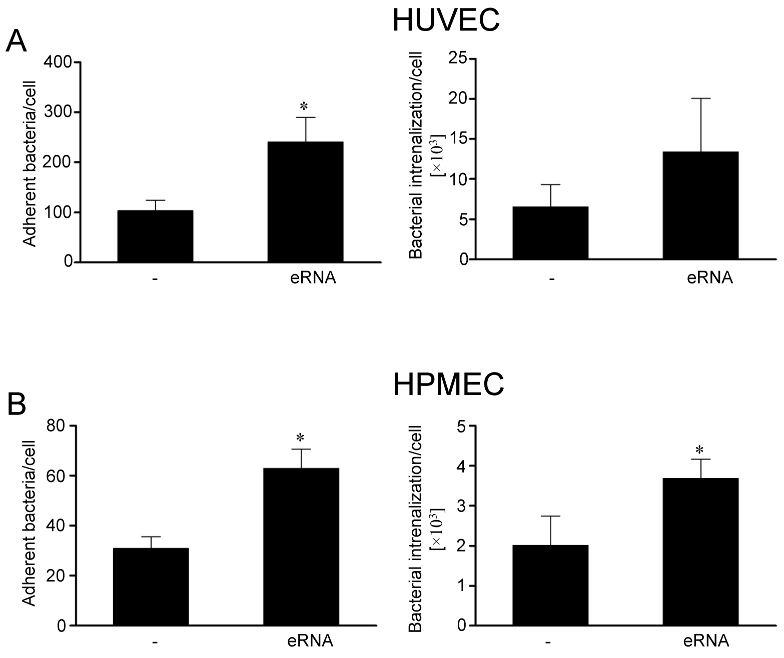

Figure S1

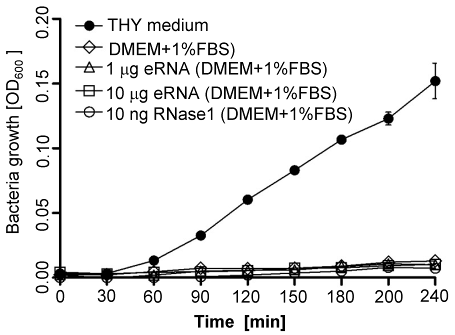
THY medium
DMEM+1%FBS)
1 μg eRNA (DMEM+1%FBS)
10 μg eRNA (DMEM+1%FBS)
10 ng RNase1 (DMEM+1%FBS)
Bacteria growth [OD600]
0.00
0.05
0.10
0.15
0.20
0
30
60
90
120
150
180
200
240
Time [min]

Figure S2

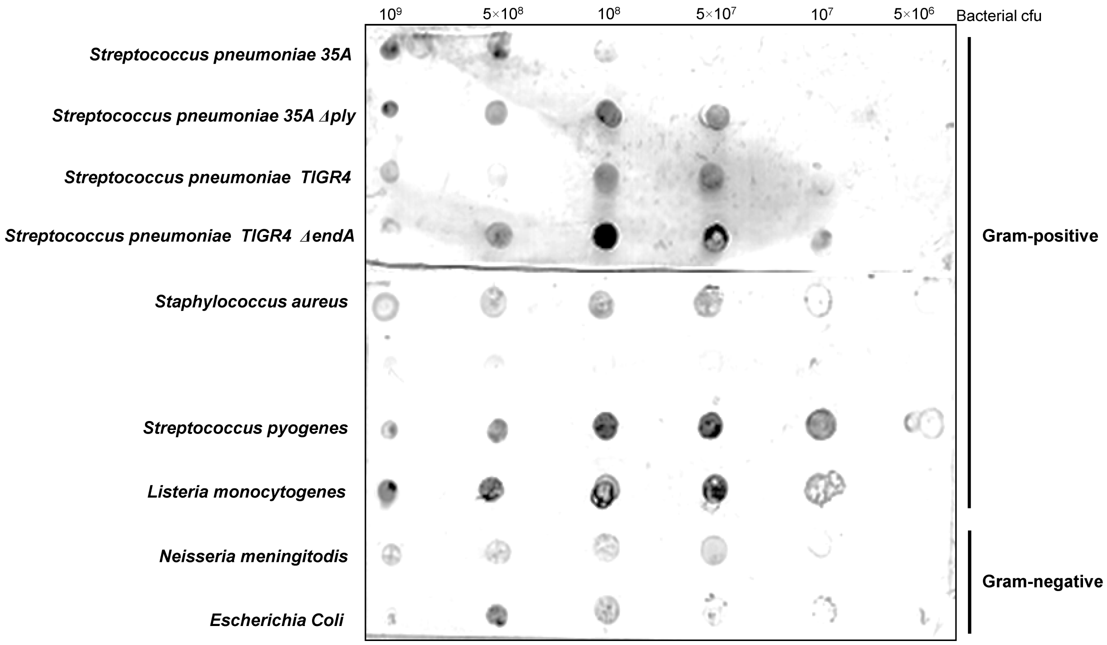
10^9
5×10^8
10^8
5×10^7
10^7
5×10^6
Bacterial cfu
Streptococcus pneumoniae 35A
Streptococcus pneumoniae 35A Δply
Streptococcus pneumoniae TIGR4
Streptococcus pneumoniae TIGR4 ΔendA
Staphylococcus aureus
Streptococcus pyogenes
Listeria monocytogenes
Neisseria meningitodis
Escherichia Coli
Gram-positive
Gram-negative

Figure S3
